# Body Mass Index and Weight Change During Adulthood Are Associated With Increased Mortality From Liver Cancer: The JACC Study

**DOI:** 10.2188/jea.JE20120199

**Published:** 2013-05-05

**Authors:** Yuanying Li, Hiroshi Yatsuya, Kazumasa Yamagishi, Kenji Wakai, Akiko Tamakoshi, Hiroyasu Iso

**Affiliations:** 1 Public Health, Department of Social and Environmental Medicine, Osaka University Graduate School of Medicine, Suita, Osaka, Japan; 1 大阪大学大学院医学系研究科社会環境医学講座公衆衛生学; 2 Department of Public Health, Fujita Health University, Toyoake, Aichi, Japan; 2 藤田保健衛生大学医学部公衆衛生学; 3 Department of Public Health Medicine, Faculty of Medicine, University of Tsukuba, Tsukuba, Ibaraki, Japan; 3 筑波大学大学院人間総合科学研究科生命システム医学専攻社会健康医学分野; 4 Department of Preventive Medicine, Nagoya University Graduate School of Medicine, Nagoya, Japan; 4 名古屋大学大学院医学系研究科健康社会医学専攻社会生命科学講座予防医学; 5 Department of Public Health, Hokkaido University Graduate School of Medicine, Sapporo, Japan; 5 北海道大学医学研究科予防医学講座公衆衛生学分野

**Keywords:** weight change, body mass index, liver cancer, mortality, prospective study, epidemiology

## Abstract

**Background:**

We investigated the association of baseline body mass index (BMI) and weight change since age 20 years with liver cancer mortality among Japanese.

**Methods:**

The data were obtained from the Japan Collaborative Cohort Study for Evaluation of Cancer Risk (JACC Study). A total of 31 018 Japanese men and 41 455 Japanese women aged 40 to 79 years who had no history of cancer were followed from 1988 through 2009.

**Results:**

During a median 19-year follow-up, 527 deaths from liver cancer (338 men, 189 women) were documented. There was no association between baseline BMI and liver cancer mortality among men or men with history of liver disease. Men without history of liver disease had multivariable hazard ratios (HR) of 1.95 (95%CI, 1.07–3.54) for BMI less than 18.5 kg/m^2^ and 1.65 (1.05–2.60) for BMI of 25 kg/m^2^ or higher, as compared with a BMI of 21.0 to 22.9 kg/m^2^. BMI was positively associated with liver cancer mortality among women and women with history of liver disease. Weight change since age 20 years was positively associated with liver cancer mortality among women regardless of history of liver disease. Women with history of liver disease had a multivariable HRs of 1.96 (1.05–3.66) for weight gain of 5.0 to 9.9 kg and 2.31 (1.18–4.49) for weight gain of 10 kg or more, as compared with weight change of −4.9 to 4.9 kg.

**Conclusions:**

Both underweight (BMI <18.5 kg/m^2^) and overweight (BMI ≥25 kg/m^2^) among men without history of liver disease, and weight gain after age 20 (weight change ≥5 kg) among women with history of liver disease, were associated with increased mortality from liver cancer.

## Abstract (ja)

**背景：:**

日本人におけるベースライン時の肥満度（BMI）及び20歳からの体重変化と肝癌死亡リスクとの関連を調べた。

**方法：:**

文部科学省科学研究費大規模コホート研究(The JACC study)のデータに基づき、年齢が40-79歳でがんの既往歴がない31,018名の日本人男性と41,455名の日本人女性を1988年から2009年まで追跡した。

**結果：:**

19年（中央値）の追跡期間中、527名（338名の男性、189名の女性）の肝癌死亡が発生した。男性全体と肝臓病の既往歴のある男性において、ベースライン時BMI値と肝癌死亡の間には関連が認められなかった。肝臓病の既往歴のない男性においては、BMI値が21.0-22.9 kg/m2の群と比べ、BMI<18.5 kg/m2群とBMI≥25 kg/m2群の肝癌死亡の多変量調整ハザード比はそれぞれ1.95 (95%CI、1.07-3.54)と1.65 (1.05-2.60)であった。女性全体と肝臓病の既往歴のある女性において、ベースライン時のBMI値と肝癌死亡との間には正の関連が認められた。女性において、20歳からの体重変化は、肝臓病の既往歴の有無にかかわらず、肝癌死亡と正の関連を示した。肝臓病の既往歴のある女性において、体重変化が−4.9-4.9 kgの群と比べ、体重増加が5.0-9.9 kgの群と10 kg以上の群の肝癌死亡の多変量調整ハザード比はそれぞれ1.96 (1.05-3.66) と2.31 (1.18-4.49)であった。

**結論：:**

肝臓病の既往歴のない男性において、低体重(BMI <18.5 kg/m2)と過体重(BMI ≥25 kg/m2)、そして、肝臓病の既往歴のある女性において、20歳からの5kg以上の体重増加は肝癌死亡のリスクと関連することが示された。

## INTRODUCTION

According to the World Health Organization, liver cancer was responsible for 700 000 deaths worldwide in 2008 and was the third leading cause of cancer death after lung cancer (1.4 million deaths) and stomach cancer (740 000 deaths).^1^

Meta-analyses^2^ and systematic reviews^3^^,^^4^ reported associations between excess body weight and higher risk of liver cancer among both men and women. However, few studies have examined the association of weight change with risk of liver cancer.^5^^,^^6^

In a population with a high prevalence of chronic infection with hepatitis C virus (HCV),^7^ it is important to determine whether body weight and weight change are associated with risk of liver cancer irrespective of viral infection (a major contributor to liver cancer).^8^ Thus, we chose to examine these associations in relation to the presence or absence of liver disease.

We conducted a prospective study of the associations of BMI at age 20 years, BMI at baseline, and weight change since age 20 years with mortality from liver cancer in a large cohort of Japanese men and women aged 40 to 79 years at baseline.

## METHODS

The Japan Collaborative Cohort Study for Evaluation of Cancer Risk (JACC study) was initiated during 1988–1990.^9^^,^^10^ Self-administered questionnaires with items on lifestyle and medical history of cancer, liver disease, gallbladder disease, diabetes mellitus, other diseases, and blood transfusion were completed by 110 588 people (46 398 men and 64 190 women) aged 40 to 79 years from 45 communities across Japan. Among them, 73 463 people (31 321 men and 42 142 women) provided self-reported data on weight and height at baseline and weight at age 20 years. We excluded 303 men and 687 women with a reported history of cancer at baseline, leaving 31 018 men and 41 455 women for the present analysis.

Mortality surveillance was conducted systematically by reviewing death certificates. Participants were followed-up until death or until they moved away from their original community, through the end of 2009 (follow-up of 4, 4, and 2 communities finished at the end of 1999, 2003, and 2008, respectively). The median follow-up period was 19.0 years. Underlying cause of death according to the International Classification of Diseases (ICD-10) was obtained centrally from the Ministry of Health and Welfare. Death from liver cancer was defined as ICD-10 codes C22.0 to C22.9. The present study was approved by the Ethical Committees of Nagoya University and Osaka University.

### Variables

Weight (kg) and height (m) were self-reported at baseline. BMI was calculated as weight (kg) divided by height (m) squared and then divided into 5 categories (<18.5, 18.5–20.9, 21.0–22.9, 23.0–24.9, and ≥25 kg/m^2^); a BMI of 21.0–22.9 kg/m^2^ served as the reference group. Weight change since age 20 years was calculated by subtracting weight at age 20 years from weight at baseline. Weight change was grouped into 5 categories (≤−10, −9.9 to −5.0, −4.9 to 4.9, 5.0 to 9.9, and ≥10 kg); stable weight (−4.9 to 4.9 kg) was used as the reference group. We asked the subjects, “Do you have a history of physician-diagnosed liver disease such as hepatitis?”. Potential confounding variables were smoking status (never, former, current smoker of <20 cigarettes per day, and current smoker of ≥20 cigarettes per day), ethanol consumption (never, former, current [1–22, 23–45, 46–68, and ≥69 g per day]), hours of walking (<0.5, 0.5, 0.6–0.9, and ≥1.0 h per day), hours of exercise (<1, 1–2, 3–4, and ≥5 per week), frequency of coffee intake (seldom, 1–2 cups per month, 1–2 cups per week, 3–4 cups per week, and almost every day), frequency of fish intake (seldom, 1–2 times per month, 1–2 times per week, 3–4 times per week, and almost every day), education level (<10, 10–12, 13–15, and ≥16 years), area of residence (Hokkaido, Tohoku, Kanto, Chubu, Kinki, Chugoku, and Kyushu regions), and histories of diabetes mellitus, gallbladder disease, and blood transfusion. A positive history of liver disease, with or without present treatment, was also considered.

### Statistical analyses

Sex-specific, age-adjusted means (SD) and proportions of potential confounding factors were calculated by a general linear model.

Cox proportional hazards models were used to calculate sex-specific age- and multivariable-adjusted hazard ratios (HRs) and 95% CIs for liver cancer mortality associated with BMI at baseline, BMI at age 20, and weight change since age 20 years. Multivariable-adjusted Cox modeling included continuous age at baseline, smoking status, ethanol consumption, hours of walking and exercise, frequencies of coffee and fish intake, education level, area of residence, and histories of diabetes mellitus, gallbladder disease, blood transfusion, and positive history of liver disease with or without present treatment. For the analysis of weight change, the model was further adjusted for height (continuous) and weight (continuous) at age 20. The *P* values for linear trends were calculated by assigning the median value of each category to corresponding individuals and treating it as a continuous variable in the model. Testing for trends was performed across the upper 3 categories of BMI (ie, ≥21.0 kg/m^2^) and weight change (>−5 kg). Testing for overall trends was performed across all 5 categories of BMI and weight change. Multivariable-adjusted HRs were also calculated for a 5-kg increment of weight change if necessary. To identify effect modification of the association between body weight or weight change and risk of liver cancer, additional stratified analyses were conducted based on the presence or absence of history of liver disease at baseline in men and women.

Because lower body weight and weight loss could be due to preclinical liver cancer, and higher body weight or weight gain could be a consequence of ascites associated with liver cancer, we excluded early deaths from liver cancer (ie, those that occurred during the first 10 years after baseline) to reduce reverse causation in our analyses.

All analyses were conducted using SAS version 9.1.3 Service Pack 4 (SAS Institute, Cary, North Carolina, USA). Two-tailed probability values of less than 0.05 were considered to indicate statistical significance.

## RESULTS

Mean age at baseline, BMI at baseline, and weight change since age 20 years, in men and women, were 57.2 (10.2) and 56.8 (9.8) years, 22.7 (2.8) and 23.0 (3.2) kg/m^2^, and 1.7 (8.9) and 2.7 (8.5) kg, respectively (Table 1). We identified 527 deaths (338 men, 189 women) from liver cancer during 1 168 909 follow-up person-years (486 745 in men, 682 164 in women).

**Table 1.** Sex-specific, age-adjusted means and proportions in all subjects and subjects with and without a self-reported history of liver disease at baseline (JACC study, 1988–2009)

	**Men**			**Women**		
	
	Total	History of liver disease^a^		Total	History of liver disease^a^	
	
		+	−		+	−
No. at risk	31 018	2438	25 793	41 455	2304	35 378
Age, years	57.2 (10.2)	58.4 (9.4)	56.6 (10.2)	56.8 (9.8)	58.9 (9.0)	56.1 (9.8)
Weight at baseline, kg	60.3 (8.8)	60.6 (9.1)	60.5 (8.8)	52.6 (7.8)	53.3 (8.5)	52.6 (7.8)
Weight at age 20, kg	58.6 (7.6)	58.4 (7.3)	58.6 (7.5)	49.9 (6.8)	49.7 (6.9)	49.9 (6.7)
Height at baseline, m	163.0 (6.6)	163.1 (6.3)	163.1 (6.6)	151.3 (5.8)	151.3 (5.7)	151.3 (5.8)
BMI at baseline, kg/m^2^	22.7 (2.8)	22.7 (2.9)	22.7 (2.8)	23.0 (3.2)	23.2 (3.3)	23.0 (3.1)
BMI at age 20, kg/m^2^	22.1 (2.8)	21.9 (2.6)	22.1 (2.7)	21.8 (3.0)	21.7 (3.0)	21.8 (3.0)
Weight change after age 20, kg	1.7 (8.9)	2.3 (9.2)	1.8 (8.7)	2.7 (8.5)	3.6 (9.1)	2.7 (8.3)
Never smoker, %	20.4	17.3	21.1	93.3	90.8	93.9
Current smoker, %	53.2	50.8	53.5	5.2	6.2	4.8
Never drinker, %	19.4	15.5	19.7	80.8	76.5	81.5
Current drinker, %	73.8	70.2	74.8	17.3	24.7	17.0
Walk 30 min or more/day, %	68.6	64.3	69.1	71.5	68.8	71.7
Exercise 3 hours or more/week, %	14.9	15.4	14.6	9.9	9.2	9.9
Coffee 1 cup or more daily, %	36.6	36.5	36.1	36.1	37.0	35.1
Fish almost daily, %	1.3	1.3	1.3	1.6	1.5	1.6
College or higher education, %	18.6	21.9	18.3	10.7	12.0	10.7
History of diabetes mellitus, %	7.1	15.9	5.3	4.0	9.4	3.1
History of gallbladder disease, %	4.3	10.7	3.2	5.6	18.9	4.1
History of blood transfusion, %	9.4	19.7	7.6	10.3	20.9	9.1
Present treatment of liver disease, %	—	28.8	—	—	22.0	—

Abbreviation: BMI, body mass index.

^a^Information on history of liver disease was missing for 2787 men and 3773 women.

Values are means (SD) or proportions.

There was no association between baseline BMI and mortality from liver cancer among men or men with liver disease. In contrast, among men without a history of liver disease the association was U-shaped: as compared with a BMI of 21.0 to 22.9 kg/m^2^, the multivariable HR (95% CI) was 1.95 (1.07–3.54) among those with a BMI less than 18.5 kg/m^2^ and 1.65 (1.05–2.60) among those with a BMI of 25 kg/m^2^ or higher. BMI was positively associated with mortality from liver cancer among women (*P* = 0.04 for overall trend) and women with a history of liver disease (*P* = 0.02 for overall trend), but not among women without a history of liver disease (*P* = 0.23 for overall trend) (Table 2).

**Table 2.** Sex-specific, age- and multivariable-adjusted hazard ratios and 95% CIs for mortality from liver cancer according to body mass index (BMI) categories at baseline (JACC study, 1988–2009)

	BMI at baseline (kg/m^2^)					*P* for trend^a^	*P* for overall trend^b^

	<18.5	18.5–20.9	21.0–22.9	23.0–24.9	≥25.0		
**Men**							
No. at risk	1669	7116	8892	7583	5758		
No. of person-years	21 231	107 857	140 791	123 112	93 753		
No. of deaths	32	82	88	73	63		
Crude death rate^c^	151	76	63	59	67		
Age-adjusted HR (95% CI)	1.87 (1.25–2.82)	1.15 (0.85–1.55)	1	1.01 (0.74–1.37)	1.20 (0.86–1.65)	0.33	0.21
Multivariable HR (95% CI)^d^	1.42 (0.93–2.15)	1.09 (0.81–1.48)	1	1.04 (0.76–1.42)	1.15 (0.83–1.60)	0.37	0.99
**Men with liver disease**							
No. at risk	139	544	690	558	507		
No. of person-years	1376	7055	9404	7940	7494		
No. of deaths	13	30	39	34	22		
Crude death rate^c^	945	425	415	428	294		
Age-adjusted HR (95% CI)	1.86 (0.99–3.51)	0.94 (0.59–1.52)	1	1.06 (0.67–1.67)	0.75 (0.44–1.26)	0.27	0.09
Multivariable HR (95% CI)^d^	0.99 (0.51–1.95)	0.91 (0.55–1.48)	1	1.03 (0.64–1.66)	0.83 (0.48–1.44)	0.38	0.80
**Men without liver disease**							
No. at risk	1303	5838	7396	6446	4810		
No. of person-years	17 660	91 245	120 182	107 098	79 876		
No. of deaths	16	39	39	33	37		
Crude death rate^c^	91	43	32	31	46		
Age-adjusted HR (95% CI)	2.24 (1.25–4.03)	1.25 (0.80–1.96)	1	1.00 (0.63–1.59)	1.58 (1.01–2.48)	0.05	0.75
Multivariable HR (95% CI)^d^	1.95 (1.07–3.54)	1.22 (0.78–1.91)	1	1.08 (0.68–1.72)	1.65 (1.05–2.60)	0.03	0.78
**Women**							
No. at risk	2560	8527	11 094	9598	9676		
No. of person-years	38 047	138 984	184 657	159 579	160 898		
No. of deaths	8	36	42	41	62		
Crude death rate^c^	21	26	23	26	39		
Age-adjusted HR (95% CI)	0.73 (0.34–1.57)	1.10 (0.71–1.72)	1	1.14 (0.74–1.75)	1.63 (1.10–2.41)	0.01	0.01
Multivariable HR (95% CI)^d^	0.74 (0.35–1.60)	1.08 (0.69–1.68)	1	1.16 (0.75–1.79)	1.42 (0.95–2.13)	0.10	0.04
**Women with liver disease**							
No. at risk	136	448	572	527	621		
No. of person-years	1722	6176	8157	7546	8749		
No. of deaths	1	16	19	17	30		
Crude death rate^c^	58	259	233	225	343		
Age-adjusted HR (95% CI)	0.23 (0.03–1.73)	1.11 (0.57–2.15)	1	1.02 (0.53–1.96)	1.49 (0.84–2.66)	0.14	0.05
Multivariable HR (95% CI)^d^	0.23 (0.03–1.74)	1.14 (0.57–2.29)	1	1.20 (0.60–2.40)	1.72 (0.93–3.18)	0.09	0.02
**Women without liver disease**							
No. at risk	2121	7282	9572	8196	8207		
No. of person-years	32 776	121 678	163 208	139 737	139 789		
No. of deaths	7	14	19	22	28		
Crude death rate^c^	21	12	12	16	20		
Age-adjusted HR (95% CI)	1.44 (0.60–3.45)	0.96 (0.48–1.92)	1	1.36 (0.74–2.52)	1.64 (0.91–2.93)	0.10	0.14
Multivariable HR (95% CI)^d^	1.32 (0.55–3.18)	0.97 (0.49–1.94)	1	1.33 (0.72–2.46)	1.49 (0.83–2.69)	0.27	0.23

^a^Test for trend refers to a baseline BMI of ≥21.0 kg/m^2^.

^b^Test for overall trend refers to a baseline BMI of <18.5 kg/m^2^.

^c^Mortality rate is expressed as rate per 100 000 person-years.

^d^Multivariable adjustment: age, smoking status, ethanol consumption, hours of walking, hours of exercise, frequencies of coffee and fish intake, education level, area of residence, histories of diabetes mellitus, gallbladder disease, blood transfusion, and positive history of liver disease with or without present treatment.

No associations were found for BMI at age 20 in either sex (Table 3). Weight change since age 20 years was positively associated with mortality from liver cancer among women, women with a history of liver disease, and women without a history of liver disease (*P* = 0.01, 0.02, and 0.03, respectively). Among women with a history of liver disease, weight gain of 5.0 to 9.9 kg was associated with a multivariable HR of 1.96 (95% CI, 1.05–3.66) for mortality from liver cancer, and weight gain of 10 kg or more was associated with an HR of 2.31 (1.18–4.49), as compared with women with a weight change of −4.9 to 4.9 kg. The multivariable HRs associated with a 5-kg increment in weight were 1.11 (1.00–1.28), 1.14 (1.00–1.30), and 1.17 (1.00–1.36) among women, women with a history of liver disease, and women without a history of liver disease, respectively. There was no association between weight change and mortality from liver cancer in men (Table 4). The results of the analyses that excluded early deaths from liver cancer were essentially identical. Among overweight men without a history of liver disease the multivariable HR was 1.67 (0.90–3.12), versus a BMI of 21.0 to 22.9 kg/m^2^. Analysis of the overall trend for BMI categories among women and women with and without a history of liver disease yielded *P* values of 0.009, 0.02, and 0.06, respectively, in the multivariable model. Analysis of overall trend for weight change in women, women with a history of liver disease, and women without a history of liver disease yielded *P* values of 0.007, 0.03, and 0.02, respectively (data not shown in table).

**Table 3.** Sex-specific, age- and multivariable-adjusted hazard ratios and 95% CIs for mortality from liver cancer according to body mass index (BMI) categories at age 20 years (JACC study, 1988–2009)

	BMI at age 20 (kg/m^2^)					*P* for trend^a^	*P* for overall trend^b^

	<18.5	18.5–20.9	21.0–22.9	23.0–24.9	≥25.0		
**Men**							
No. at risk	1805	9180	10 300	6372	3361		
No. of person-years	28 079	148 241	164 069	97 587	48 768		
No. of deaths	14	91	115	75	43		
Crude death rate^c^	50	61	70	77	88		
Age-adjusted HR (95% CI)	0.78 (0.45–1.36)	0.99 (0.75–1.30)	1	0.96 (0.72–1.29)	1.02 (0.72–1.45)	0.92	0.65
Multivariable HR (95% CI)^d^	0.74 (0.42–1.29)	0.89 (0.68–1.18)	1	0.92 (0.69–1.24)	0.91 (0.64–1.31)	0.54	0.66
**Men with liver disease**							
No. at risk	170	735	785	474	274		
No. of person-years	2249	10 227	11 072	6248	3474		
No. of deaths	8	34	48	31	17		
Crude death rate^c^	356	332	434	496	489		
Age-adjusted HR (95% CI)	0.86 (0.41–1.82)	0.83 (0.53–1.28)	1	1.02 (0.65–1.61)	0.97 (0.55–1.69)	0.98	0.49
Multivariable HR (95% CI)^d^	0.91 (0.42–1.97)	0.68 (0.43–1.07)	1	0.98 (0.61–1.56)	0.75 (0.41–1.35)	0.36	0.68
**Men without liver disease**							
No. at risk	1456	7667	8625	5294	2751		
No. of person-years	23 539	127 328	141 043	83 287	40 864		
No. of deaths	4	48	57	33	22		
Crude death rate^c^	17	38	40	40	54		
Age-adjusted HR (95% CI)	0.78 (0.45–1.36)	0.99 (0.75–1.30)	1	0.96 (0.72–1.29)	1.02 (0.72–1.45)	0.85	0.61
Multivariable HR (95% CI)^d^	0.50 (0.18–1.39)	1.05 (0.71–1.54)	1	0.81 (0.53–1.25)	0.98 (0.60–1.62)	0.76	0.99
**Women**							
No. at risk	4061	12 936	11 996	7364	5098		
No. of person-years	64 811	214 456	199 655	122 099	81 143		
No. of deaths	20	49	58	37	25		
Crude death rate^c^	31	23	29	30	31		
Age-adjusted HR (95% CI)	1.23 (0.74–2.05)	0.90 (0.61–1.32)	1	0.95 (0.63–1.43)	0.77 (0.48–1.23)	0.28	0.25
Multivariable HR (95% CI)^d^	0.98 (0.58–1.64)	0.85 (0.58–1.25)	1	0.91 (0.60–1.38)	0.73 (0.45–1.18)	0.18	0.49
**Women with liver disease**							
No. at risk	289	721	582	410	302		
No. of person-years	3746	10 014	8348	6049	4192		
No. of deaths	11	17	28	13	14		
Crude death rate^c^	294	170	335	215	334		
Age-adjusted HR (95% CI)	1.04 (0.52–2.10)	0.56 (0.31–1.02)	1	0.64 (0.33–1.24)	0.84 (0.44–1.60)	0.60	0.99
Multivariable HR (95% CI)^d^	0.91 (0.43–1.94)	0.53 (0.29–1.00)	1	0.61 (0.31–1.21)	0.75 (0.37–1.50)	0.32	0.98
**Women without liver disease**							
No. at risk	3337	11 081	10 396	6311	4253		
No. of person-years	55 240	188 811	176 982	106 855	69 299		
No. of deaths	9	27	25	20	9		
Crude death rate^c^	16	14	14	19	13		
Age-adjusted HR (95% CI)	1.23 (0.74–2.05)	0.90 (0.61–1.32)	1	0.95 (0.63–1.43)	0.77 (0.48–1.23)	0.25	0.14
Multivariable HR (95% CI)^d^	1.28 (0.59–2.76)	1.24 (0.72–2.15)	1	1.22 (0.68–2.21)	0.62 (0.29–1.34)	0.30	0.13

^a^Test for trend refers to a baseline BMI of ≥21.0 kg/m^2^.

^b^Test for overall trend refers to a baseline BMI of <18.5 kg/m^2^.

^c^Mortality rate is expressed as rate per 100 000 person-years.

^d^Multivariable adjustment: age, smoking status, ethanol consumption, hours of walking, hours of exercise, frequencies of coffee and fish intake, education level, area of residence, histories of diabetes mellitus, gallbladder disease, blood transfusion, and positive history of liver disease with or without present treatment.

**Table 4.** Sex-specific, age- and multivariable-adjusted hazard ratios and 95% CIs for mortality from liver cancer according to categories of weight change since age 20 years to baseline (JACC study, 1988–2009)

	Weight change from age 20 to baseline (kg)					*P* for trend^a^	*P* for overall trend^b^

	≤−10.0	−5 to −9.9	−4.9 to 4.9	5.0 to 9.9	≥10.0		
**Men**							
No. at risk	2454	4831	12 279	5666	5788		
No. of person-years	30 812	70 268	197 516	93 608	94 540		
No. of deaths	27	76	124	55	56		
Crude death rate^c^	88	108	63	59	59		
Age-adjusted HR (95% CI)	0.94 (0.61–1.43)	1.32 (0.99–1.77)	1	1.06 (0.77–1.46)	1.09 (0.80–1.50)	0.59	0.85
Multivariable HR (95% CI)^d^	0.68 (0.43–1.08)	1.08 (0.80–1.46)	1	1.06 (0.77–1.47)	0.98 (0.70–1.37)	0.88	0.54
**Men with liver disease**							
No. at risk	193	390	882	436	537		
No. of person-years	1942	4895	12 357	6322	7752		
No. of deaths	11	29	49	23	26		
Crude death rate^c^	566	592	397	364	335		
Age-adjusted HR (95% CI)	1.09 (0.56–2.12)	1.26 (0.79–2.00)	1	1.03 (0.62–1.69)	0.97 (0.60–1.56)	0.89	0.48
Multivariable HR (95% CI)^d^	0.59 (0.28–1.23)	0.93 (0.56–1.54)	1	1.08 (0.64–1.81)	1.03 (0.61–1.72)	0.63	0.33
**Men without liver disease**							
No. at risk	1908	3880	10 406	4818	4781		
No. of person-years	24 969	58 307	171 105	81 354	80 327		
No. of deaths	13	33	65	28	25		
Crude death rate^c^	52	57	38	34	31		
Age-adjusted HR (95% CI)	0.95 (0.52–1.73)	1.15 (0.75–1.76)	1	1.03 (0.66–1.61)	0.94 (0.59–1.50)	0.84	0.73
Multivariable HR (95% CI)^d^	0.69 (0.36–1.34)	0.98 (0.64–1.52)	1	1.08 (0.69–1.70)	1.00 (0.61–1.61)	0.95	0.52
**Women**							
No. at risk	2269	5811	16 186	8986	8203		
No. of person-years	32 934	92 910	270 774	150 616	134 929		
No. of deaths	10	24	62	46	47		
Crude death rate^c^	30	26	23	31	35		
Age-adjusted HR (95% CI)	0.77 (0.39–1.52)	0.82 (0.51–1.32)	1	1.44 (0.98–2.10)	1.60 (1.10–2.34)	0.01	0.0006
Multivariable HR (95% CI)^d^	0.68 (0.34–1.40)	0.83 (0.51–1.35)	1	1.31 (0.89–1.94)	1.41 (0.94–2.11)	0.08	0.01
**Women with liver disease**							
No. at risk	135	308	789	508	564		
No. of person-years	1750	4400	11 184	7488	7528		
No. of deaths	3	15	21	22	22		
Crude death rate^c^	171	341	188	294	292		
Age-adjusted HR (95% CI)	0.67 (0.20–2.26)	1.56 (0.80–3.04)	1	1.64 (0.90–2.98)	1.70 (0.94–3.10)	0.06	0.11
Multivariable HR (95% CI)^d^	0.55 (0.15–2.03)	1.47 (0.72–3.00)	1	1.96 (1.05–3.66)	2.31 (1.18–4.49)	0.02	0.02
**Women without liver disease**							
No. at risk	1812	4880	14 141	7720	6825		
No. of person-years	27 205	79 851	241 627	132 498	116 006		
No. of deaths	4	6	36	22	22		
Crude death rate^c^	15	8	15	17	19		
Age-adjusted HR (95% CI)	0.54 (0.19–1.52)	0.35 (0.15–0.84)	1	1.22 (0.72–2.08)	1.36 (0.80–2.30)	0.24	0.003
Multivariable HR (95% CI)^d^	0.50 (0.17–1.49)	0.35 (0.15–0.85)	1	1.16 (0.68–1.99)	1.14 (0.65–2.00)	0.58	0.03

^a^Test for trend refers to weight change ≥−4.9 kg.

^b^Test for overall trend refers to weight change ≤−10.0 kg.

^c^Mortality rate is expressed as rate per 100 000 person-years.

^d^Multivariable adjustment: age, weight at age 20, height at age 20, smoking status, ethanol consumption, hours of walking, hours of exercise, frequencies of coffee and fish intake, education level, area of residence, histories of diabetes mellitus, gallbladder disease, blood transfusion, and positive history of liver disease with or without present treatment.

## DISCUSSION

In this large-scale prospective study of Japanese men and women, we observed that overweight and underweight were associated with liver cancer mortality in men without liver disease and that weight change positively correlated with liver cancer mortality in women, regardless of history of liver disease.

Our results showing an excess risk of mortality from liver cancer in overweight men without a history of liver disease are in line with those from studies of men from the general populations of East Asian countries,^11^ European countries,^5^^,^^6^ and the United States.^12^ Among women, we found a weak positive association between BMI categories and liver cancer mortality, which supports previous findings for women from the general populations of the United States^12^ and Korea.^11^ The positive association between BMI and liver cancer risk in women with a history of liver disease was in line with previous findings in patients with liver disease, namely, that a higher baseline BMI was predictive of incident liver cancer.^13^^–^^19^

Two prospective studies investigated the association of weight change during adulthood with liver cancer risk.^5^^,^^6^ A study of 107 815 Swedish men with a small number of incident liver cancers (*n* = 55) reported no association between weight gain and risk of liver cancer, as compared with stable weight.^5^ Another study of 191 927 European men and women found a positive dose-dependent association between weight change after age 20 years and risk of incident liver cancer.^6^ However, in sex-specific analysis, there was a positive association only among men, perhaps due to the small number of incident liver cancers among women (*n* = 54). Nonetheless, we found a positive relationship between weight change and liver cancer mortality in women, regardless of history of liver disease, which confirms previous findings among men. Because the numbers of deaths were relatively small in the first 2 weight-change groups (ie, ≤−10.0 and −5 to −9.9 kg) in the analyses of women in the present study, we examined whether combining the first 2 weight-change groups would alter the results; however, the *P* values were very similar for overall trend. We found no association between weight change since age 20 years and risk of liver cancer in men.

The mechanism linking excess body weight or weight gain during adulthood with higher mortality from liver cancer may be mediated via progression of nonalcoholic fatty liver disease (NAFLD), a clinicopathologic condition that encompasses a wide spectrum of liver tissue changes, ranging from steatosis alone to nonalcoholic steatohepatitis, advanced fibrosis, cirrhosis, and, in the most severe cases, liver cancer.^20^ Level of obesity was found to be correlated with NAFLD development: a study of 39 151 Japanese adults reported that 12.8% of nonobese subjects (BMI <25 kg/m^2^), 51.4% of overweight subjects (25 ≤ BMI < 30 kg/m^2^), and 80.4% of highly obese subjects (BMI ≥30 kg/m^2^) had fatty liver disease, as determined by abdominal ultrasonography.^21^ In addition, weight gain during an average of 414 days was found to be an independent risk factor for incident NAFLD in Japanese men and women.^22^

Second, overweight (BMI ≥25 kg/m^2^) was associated with a 5-fold risk of fibrosis progression in liver during a 1-year period among people with HCV infection,^23^ which suggests that overweight increases the risk of liver cancer via progression of liver fibrosis.

Third, it is possible that overweight was confounded by hepatitis C infection. However, according to a nested case-control study^24^ of a JACC study subsample of approximately 12 000 adults, BMI tended to be inversely associated with HCV infection. For example, among men without liver disease, the proportion of those with HCV infection was 8.9% for a BMI less than 18.5 kg/m^2^, 7.3% for a BMI of 18.5 to less than 21.0 kg/m^2^, 6.9% for a BMI of 21.0 to less than 23.0 kg/m^2^, 6.7% for a BMI of 23.0 to less than 25.0 kg/m^2^, and 4.7% for a BMI of 25.0 kg/m^2^ or higher. Thus, it is unlikely that the excess risk of mortality from liver cancer in adults with a BMI of 25.0 kg/m^2^ or higher was due to HCV infection. However, the excess risk of mortality from liver cancer in those with a BMI less than 18.5 kg/m^2^ could be confounded by HCV infection.

In the present study, men without liver disease and a baseline BMI less than 18.5 kg/m^2^ had excess mortality from liver cancer as compared with those with a BMI of 21.0 to 22.9 kg/m^2^, perhaps because underweight men without liver disease were in a preclinical disease state. Indeed, in our study the proportion of former drinkers was higher among underweight men without liver disease than among men with a BMI of 21.0 to 22.9 kg/m^2^ (10% vs 5%). However, the associations were weaker in sensitivity analyses that excluded deaths from liver cancer within 10 years (*n* = 77) and former drinkers (*n* = 1277) from men without liver disease: the multivariable HRs were 1.80 (95% CI, 0.72–4.53) and 1.86 (95% CI, 0.96–3.58), respectively. Therefore, the increased risk of mortality from liver cancer associated with low BMI is unlikely to be due to reverse causation. The mechanisms responsible should be investigated in future studies.

Our study benefited from a long follow-up period and a large population-based sample, which allowed us to examine associations with liver cancer in narrow ranges of BMI and weight change in both men and women. However, some limitations of the present study should be discussed. First, the lack of information on HCV infection, a major risk factor for liver cancer,^8^ is a major limitation of the current study. Because most individuals with hepatitis virus infection are asymptomatic, the use of a questionnaire to exclude hepatitis would be insufficient. Second, we used mortality data rather than incidence data as an endpoint. However, the prognosis of liver cancer is generally poor: relative 5-year survival rates were 21.2% to 27.1% from 1993–1996 to 2000–2002, according to statistics by the Japan National Cancer Center,^25^ which means that most incident cases are detected as mortality cases.^25^ Third, weight and height were self-reported in the current study and were not validated by actual measurements. However, a previous validation study of a Japanese population indicated that self-reported weight and height strongly correlated with previously measured weight and height: the reported Pearson correlation coefficients for men and women were 0.979 and 0.998 for height and 0.961 and 0.959 for weight, respectively, ie, the differences were immaterial.^26^ Fourth, weight at age 20 was also self-reported. However, 1 study found that long-term recall of past body weights was reasonably accurate in Japanese adults^27^ and that people who had experienced weight loss after age 25 years underestimated their past weights, whereas those with stable weight or weight gain overestimated them.^27^ Recall bias resulting in misclassification of weight changes would likely lead to overestimation of real associations. Fifth, only 70% of the present cases reported their weight at age 20 and at baseline. However, any selection bias caused by missing data is unlikely to affect the results because age (57.0 vs 58.2 years), baseline BMI (22.8 vs 22.7 kg/m^2^), and other baseline variables were similar between the included and excluded subjects.

In conclusion, underweight (BMI <18.5 kg/m^2^) and overweight (BMI ≥25 kg/m^2^) in men without liver disease, and weight gain (weight change ≥5 kg) after age 20 in women with liver disease, were associated with increased mortality from liver cancer. Higher BMI tended to be associated with higher mortality from liver cancer among women and women with a history of liver disease. Weight change was positively associated with increased risk of liver cancer mortality in women with or without liver disease.

## ONLINE ONLY MATERIALS

Abstract in Japanese.
