# Supplementary material for: Body Mass Index and Weight Change During Adulthood Are Associated With Increased Mortality From Liver Cancer: The JACC Study
Source: J Epidemiol. 2013 May 5;23(3):219–26. doi: 10.2188/jea.JE20120199 (PMC3700251; doi:10.2188/jea.JE20120199)
Supplement: Abstract in Japanese. [file je-23-219-s001.pdf]

成年期の肥満度及び体重変化は肝癌死亡リスクと関連する（The JACC Study）

李　媛英[1]、八谷　寛[2]、山岸　良匡[3]、若井　建志[4]、玉腰　暁子[5]、磯　博康[1]

1. 大阪大学大学院医学系研究科社会環境医学講座公衆衛生学
2. 藤田保健衛生大学医学部公衆衛生学
3. 筑波大学大学院人間総合科学研究科生命システム医学専攻社会健康医学分野
4. 名古屋大学大学院医学系研究科健康社会医学専攻社会生命科学講座予防医学
5. 北海道大学医学研究科予防医学講座公衆衛生学分野

背景：日本人におけるベースライン時の肥満度（BMI）及び20歳からの体重変化と肝癌死亡リスクとの関連を調べた。

方法：文部科学省科学研究費大規模コホート研究(The JACC study)のデータに基づき、年齢が40-79歳でがんの既往歴がない31,018名の日本人男性と41,455名の日本人女性を1988年から2009年まで追跡した。

結果： 19年（中央値）の追跡期間中、527名（338名の男性、189名の女性）の肝癌死亡が発生した。男性全体と肝臓病の既往歴のある男性において、ベースライン時BMI値と肝癌死亡の間には関連が認められなかった。肝臓病の既往歴のない男性においては、BMI値が21.0-22.9 kg/m^{2}の群と比べ、BMI<18.5 kg/m^{2}群とBMI≥25 kg/m^{2}群の肝癌死亡の多変量調整ハザード比はそれぞれ1.95 (95%CI、1.07-3.54)と1.65 (1.05-2.60)であった。女性全体と肝臓病の既往歴のある女性において、ベースライン時のBMI値と肝癌死亡との間には正の関連が認められた。女性において、20歳からの体重変化は、肝臓病の既往歴の有無にかかわらず、肝癌死亡と正の関連を示した。肝臓病の既往歴のある女性において、体重変化が—4.9-4.9 kgの群と比べ、体重増加が5.0=9.9 kgの群と10 kg以上の群の肝癌死亡の多変量調整ハザード比はそれぞれ1.96 (1.05-3.66) と2.31 (1.18-4.49)であった。

結論：肝臓病の既往歴のない男性において、低体重(BMI <18.5 kg/m^2)と過体重(BMI ≥25 kg/m^2)、そして、肝臓病の既往歴のある女性において、20歳からの5kg以上の体重増加は肝癌死亡のリスクと関連することが示された。

キーワード：体重変化、肥満度、肝癌、死亡率、前向き研究、疫学
